# The Challenge of CRISPR-Cas Toward Bioethics

**DOI:** 10.3389/fmicb.2021.657981

**Published:** 2021-05-28

**Authors:** Luis Uriel Gonzalez-Avila, Juan Manuel Vega-López, Leda Ivonne Pelcastre-Rodríguez, Omar Alejandro Cabrero-Martínez, Cecilia Hernández-Cortez, Graciela Castro-Escarpulli

**Affiliations:** ^1^Laboratorio de Investigación Clínica y Ambiental, Departamento de Microbiología, Escuela Nacional de Ciencias Biológicas, Instituto Politécnico Nacional, Ciudad de Mexico, Mexico; ^2^Laboratorio de Bioquímica Microbiana, Departamento de Microbiología, Escuela Nacional de Ciencias Biológicas, Instituto Politécnico Nacional, Ciudad de Mexico, Mexico

**Keywords:** CRISPR-Cas, bioethics, laws, genetic, biotechnology

## Abstract

Since determining the structure of the DNA double helix, the study of genes and genomes has revolutionized contemporary science; with the decoding of the human genome, new findings have been achieved, including the ability that humans have developed to modify genetic sequences *in vitro*. The discovery of gene modification mechanisms, such as the CRISPR-Cas system (Clustered Regularly Interspaced Short Palindromic Repeats) and Cas (CRISPR associated). Derived from the latest discoveries in genetics, the idea that science has no limits has exploded. However, improvements in genetic engineering allowed access to new possibilities to save lives or generate new treatment options for diseases that are not treatable by using genes and their modification in the genome. With this greater knowledge, the immediate question is who governs the limits of genetic science? The first answer would be the intervention of a legislative branch, with adequate scientific advice, from which the logical answer, bioethics, should result. This term was introduced for the first time by Van Rensselaer Potter, who in 1970 combined the Greek words bios and ethos, *Bio-Ethik*, which determined the study of the morality of human behavior in science. The approach to this term was introduced to avoid the natural tension that results from the scientific technical development and the ethics of limits. Therefore, associating the use of biotechnology through the CRISPR-Cas system and the regulation through bioethics, aims to monitor the use of techniques and technology, with benefits for humanity, without altering fundamental rights, acting with moral and ethical principles.

## Introduction

Since the discovery of the DNA structure described by Watson and Crick in 1953, the generation of knowledge about the molecular genetic bases began. It was determined that the double helix contained all the genetic information of the individuals made up of the four bases, adenine, thymine, guanine, and cytosine. After this event, in the field of genetics, the human genome was sequenced, discovering that it is made up of 3 billion base pairs, which oversees the production of millions of different proteins with the help of the complex cellular system, which allows the body to function. These advances have allowed the development of gene therapy, through which it is sought to interfere in gene expression through corrective manipulation based on sequence cutting and pasting techniques (Pérez, 2006; Yabar, 2019; Espinosa and Hernández-Hernández, 2020).

Advances in genetic engineering have been advancing, and proposals for innovations and simplification of techniques, as more details about DNA are known, allow the study and understanding of the complex genomic system of expression and the shutdown of the genes. Molecular techniques have sought to correct the damage in the sequence of the carrier of a disease encoded in the genome; however, they have not achieved their objective, since there is no absolute control over the damage that can occur in the carrier, trying to prevent it from the damage inherited to the offspring. The use of these techniques involves diverse and complex techniques *in vivo* and *in vitro*, the mechanisms used are mainly based on the use of vectors that seek to introduce a specific or modified gene, which is capable of being transcribed and the mRNA is produced to be translated (You et al., 2019; Jamal et al., 2020).

The three principles of bioethics initially proposed in the Belmont report in 1978, were beneficence, autonomy of patients, and justice. Later, in the work of Beauchamp and Childres Principles of Biomedical Ethics, they added the fourth principle, which they called non-maleficence. These principles that obey a marinist philosophical reflection, which was initially promoted by the National Commission for the Protection of Human Subjects of Biomedical and Behavioral Research, constituted the study of ethical issues related to biomedical research (Gómez-Tatay and Aznar, 2019; Shkomova, 2019; Schupmann et al., 2020).

In this concept, non-maleficence, would highlight the premise of *Primum non nocere*, translated as “first, do no harm,” based upon the studies on the corresponding criteria. Avoiding the improper use of the sequences, especially the molecular biology techniques that are used for this. Those that use restriction enzymes, cloning of sequences in plasmids, integrons, the use of nucleases or the recently described CRISPR Cas, which, since its discovery, it generated interest in the scientific community due to the rarity and complexity of the system, contemporary medicine, and even technology are not allowed (Capella, 2016; Cribbs and Perera, 2017; Noll, 2019).

## CRISPR-Cas Overview

The acronym CRISPR comes from Clustered Regularly Interspaced Short Palindromic Repeats, and the second part Cas refers to nuclease-like proteins that are associated with the CRISPR system (CRISPR associated system) (Capella, 2016; Hille et al., 2018).

The first CRISPR-Cas systems being detected over 30 years ago in *Escherichia coli* (Ishino et al., 1987) and with the acronym of the system being defined on the early 2000s (Jansen et al., 2002); the overall study of these systems has become widely popular due to their properties and multiple applications. The CRISPR-Cas genomic loci consist of a CRISPR array composed of direct repeats with unique spacers between them and the Cas genes, the number of these arrays that one genome can harbor ranges from 1 to 18, while the number of repeat units in one array ranges from 2 to 374 (Marraffini and Sontheimer, 2010).

These systems, more widely known as genome engineering tools, achieve immunity by incorporating fragments of foreign nucleic acids into the CRISPR arrays, enabling a series of proteins to sense by base-pair complementarity to perform the cleavage of the specific DNA or RNA sequences from the exogenic elements (Makarova et al., 2020).

The immune response creates and keeps updating a molecular file of encounters with foreign nucleic acids in the form of spacers; sequences of typically 32–38 nucleotides (nt) of length, ranging from 21 to 72 nt (Barrangou and Marraffini, 2014). These spacers are subsequently used to protect the bacterium or archaeon against new infections with a similar agent (Faure et al., 2019). Acquired spacers in the adaptation stage are then transcribed and processed into crRNAs (CRISPR RNAs) in the expression stage to allow the start of the final interference stage, in which the crRNA is used to recognize the complementary or partially complementary sequence of the spacer present in the invading mobile genetic element (MGE), this is followed by the cleavage and inactivation of said element by either one or more Cas nucleases (Faure et al., 2019).

The defensive strategy consists of the generation of the guide RNA that is an exact copy of the viral DNA, said RNA sequence will function as a guide for the Cas protein for the identification of the genetic material of the virus. By means of the complementary sequences of DNA and gRNA, they hybridize, concluding with the cut made by the Cas protein for viral inactivation (Mojica et al., 2005).

In general, the operation can be understood in three phases; the first consists of adaptation, in which foreign genetic material is incorporated into the locus spacers to save said sequences for future attacks. The incorporation of these sequences is carried out by horizontal gene transfer, degrading the foreign DNA, leaving it as new spacers. The second phase consists of the transcription of the CRISPR Cas, generating a precursor (CRISPR-RNA or pre-crRNA), which is processed and generates the crRNAs that are complementary to the foreign DNA sequence. In the third phase, commonly known as interference, the Cas proteins, using guide crRNAs, detect foreign sequences and degrade them (Makarova et al., 2015; Capella, 2016; Mojica and Rodriguez-Valera, 2016).

**FIGURE 1:**
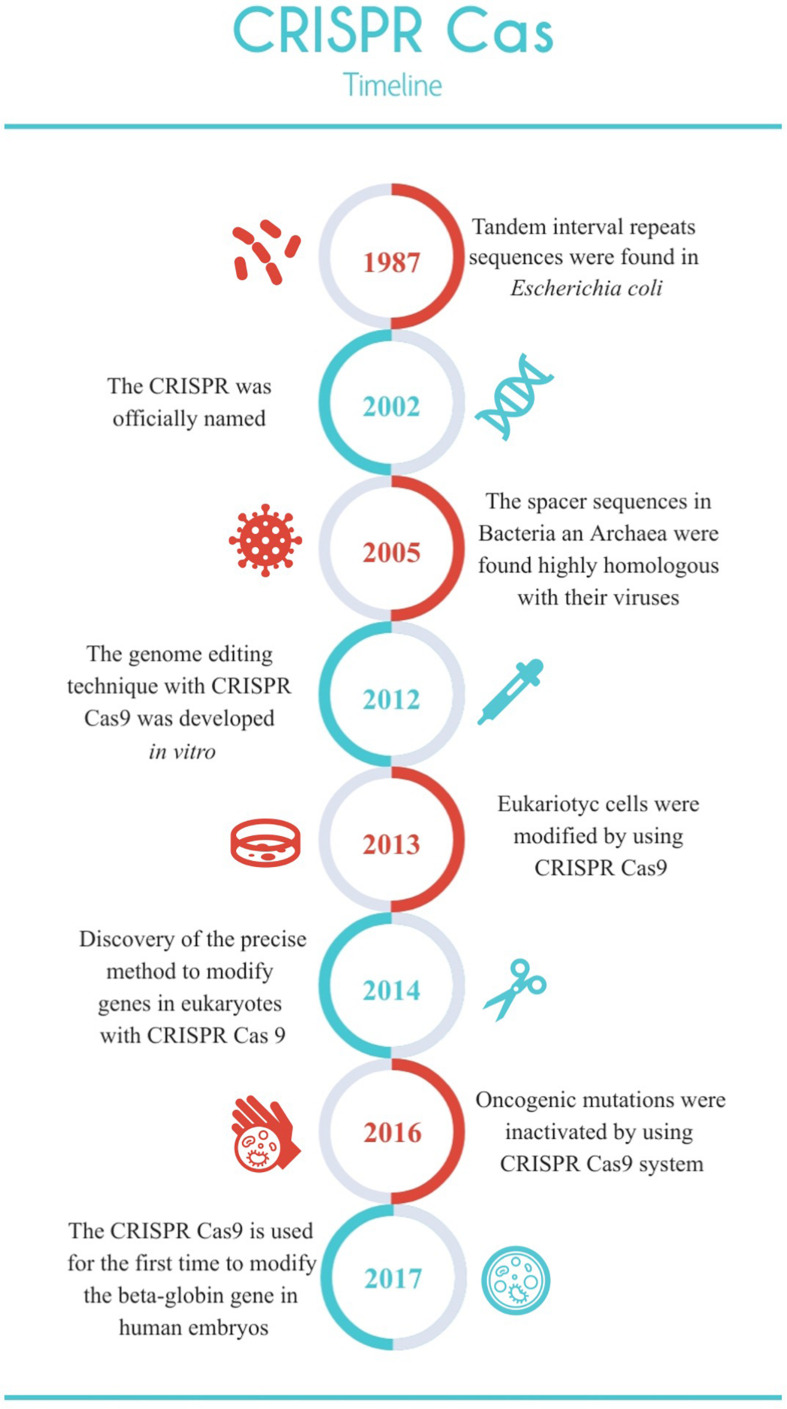
Timeline of the most relevant discoveries about the CRISPR Cas system.

The CRISPR revolution has been made possible by the identification of the right enzymatic systems that simplify methodologies to exploit the potential of CRISPR-Cas systems, in a similar fashion to the development of the polymerase chain reaction (PCR) (Ishino et al., 2018). Due to the complexity and potential biotechnological application, research has increased exponentially, allowing studies on the genetic manipulation of species, modification of cell lines, and the creation of new mutants (Figure 1). One of the most important is the genetic modification of patients affected by a disease, but who sets the limits for these scientific advances? (Caballero-Hernández et al., 2017; Hirsch et al., 2019).

### CRISPR-Cas Technology Advancements

Science from its conception tries to generate knowledge and discoveries that allow a benefit for humanity. The possibility of having a cure for almost any genetically based disease is undeniable, however, as in all stories, there is a good and a bad part. The bad part usually has the economic interests of large companies added (Wu et al., 2020).

Information on the application of the CRISPR Cas system should focus on deeply studying the damages or collateral effects of said system, when using it in living beings, and finally in humans. The feasibility of the target sites that are intended to be modified to solve, in general, a health problem, should be carefully studied in cell culture models, possibly also in animal models, waiting for favorable results, without the alteration of other metabolic factors. Derived from the growing interest in the use and application of CRISPR Cas for genetic modification, the scientific community is often in ethical dilemmas, due to the division of ideas that, on the one hand, promise encouraging results, and on the other hand, there is the question of commitment of life in general (Cai and Wang, 2019; Pickar-Oliver and Gersbach, 2019; Wu et al., 2020).

Ethical dilemmas and the need for a body that guarantees the rights of living beings subjected to biomedical processes gave rise to bioethics, whose main task would be to ensure human rights and dictate the principles that ensure respect for life, in other words, stipulates the bases on which the investigations will be developed, how researchers should be governed and the bases of their investigations (Cribbs and Perera, 2017; Marinelli and Del Rio, 2020).

In 2014, Zhang’s group and collaborators from the Broad Institute obtained a patent that granted them the right to use the CRISPR system in mice, humans, pigs, and almost any organism, other than bacteria. First controversial aspect on the use of this tool, is that the patent was obtained quickly, in less than six months. Furthermore, the works of Doudna and Charpentier, who had previously applied for a patent, had been rejected, as their possible use in humans had been speculated, contrary to what was published by Zhang, who had already tested it in humans, and had been in place since 2012 (Cong et al., 2013).

Nevertheless, in 2011, they had begun collaborative studies with the group of Doudna and Charpentier, who unified knowledge about *Streptococcus pyogenes* and RNA, respectively. The association of the two researchers allowed the beginning of the era of <genomic scissors>, together with the standardization of the *in vitro* method. The enzyme used for this purpose is the Cas 9 protein that acts on DNA through the guidance of RNA, forming a chimera. The simplification of the method allowed the conversion of a natural phenomenon into a genetic engineering technique. In such a way that this advance would allow the use of the system for gene editing in eukaryotic cells in a specific and precise way in predetermined sites, and not only providing immunity to bacteria (Doudna and Charpentier, 2014).

Later, Zhang’s group, in 2017, gave a twist to the CRISPR Cas technology through its publication in which the ability to edit RNA by using the Cas 13 protein was presented, associating it with the adenosine deaminases protein (ADAR). The editing system was called REPAIR (RNA Editing for Programmable Adenosine to Inosine Replacement), this new technology would allow to change an adenosine base with a base inosine, to correct the point mutations that cause genetic diseases due to defects in the RNA. Subsequently, they sought to correct with an efficiency of 20–40%, experimenting with *in vitro* mutations of conditions, such as Fanconi anemia or nephrogenic diabetes insipidus, correcting them successfully by using the REPAIR system (D’Souza, 2017; Gootenberg et al., 2017).

Some variants of the CRISPR Cas system have been used for genome editing, due to the efficiency of gene editing and the wide scope of genome orientation, of which the Cas9 protein is the most widely used, of which, various investigations have focused in modifying the Cas9 protein and increasing its efficiency. The applications are diverse, epigenetic editing is listed for the specific alteration of loci, regulation of genes for the activation or deactivation of the expression of a gene or group of genes. The monitoring of cell dynamics by chromatin analysis in conjunction with the 3D modification of cell chromatin. By allowing DNA recognition and RNA editing, the application of the system is enormous, from biomedicine to biotechnology, some examples of the current CRISPR-Cas systems application related to control or to cure diseases are focused on multiple myeloma; esophageal, lung, prostate, and bladder cancer; solid tumors; melanoma; leukemia; human papilloma virus; HIV-1; gastrointestinal infection; β-thalassemia; sickle-cell anemia, among others (Brokowski and Adli, 2019).

Various investigations can be counted, however, each one of them has points that can be subjected to deep discussion by a scientific committee that evaluates and, where appropriate, approves or rejects them. This is due to the fact that the total effectiveness is still being observed and without adverse effects, that is, in the trial period. The doubt is that the Cas 9 protein has not shown a 100% effectiveness, since it has a relatively frequent variability of cut, so there is a diversity of modified sequences and some with some similarity to other mutations, which could generate adverse effects (Bhan et al., 2017; Caballero-Hernández et al., 2017).

Due to the lack of confidence in the CRISPR Cas system and its application in the cure of genetic diseases, voices have arisen claiming to be careful with releasing them, in addition to the fact that few or perhaps none of the countries are already thinking about laws that regulate the use of these techniques, in addition to the probable complication of patent application. The question arises as to whether the modified genetic sequences are patentable (Capella, 2016; Bhan et al., 2017).

### The Limits of Genetic Modification

In the broad field of genetic engineering research, the number of laboratories with scientists dedicated to research this system has increased in recent years. Probably each one of them has proposed to solve a problem through this genetic technology, which will eventually fall into the patent fight. Moreover, the society demands the release of this technique to save lives worldwide, claiming that this discovery cannot be under a patent, since it would become almost or totally unattainable for many human beings; however, scientists warn about its release (Cribbs and Perera, 2017; Khan, 2019).

All over the world, genetic modification has been an object of reflection for bioethics, and this has been increased by the arrival, in large part, of the CRISPR Cas system. It is important to note that technology of CRISPR Cas is not inherently “good” or “bad,” technology is tools and forms of power, which can be well illustrated by Michael Foucalt in his concept of biopolitics and the implementation of power over our bodies. In such a way that, the result of the use of new genetic modification technologies can be something applauded or something deplorable, taking into account that the tool used is not the one that determines the end, it is the user who determines the fact. This technology and the stem cell modification line represent a great potential for the development of revolutionary genetic therapies, representing a feasible possibility of exploitation and clinical application. After the probable approval of CRISPR Cas as a therapeutic alternative, it is questioned how feasible it is to approve it, if it will be accessible to the public, in which cell lines it could be applied, in addition to the laws that should govern its use. The origin of the publications that have genetically modified human embryos by using CRISPR Cas 9, has caused different scientists to pronounce on the location or suspension of this type of research, however, these pronouncements should be accompanied by arguments on how to regulate these novel genetic tools (Brokowski, 2018; Lee and Kim, 2018).

When questioning to what extent the use of CRISPR in clinical medicine should be allowed, through the use of autonomy, it must clarify whether the user truly knows the risk of undergoing these treatments. Here, morality and what is legally permissible is considered, seeking to justify the use without considering the risk posed by research involving CRISPR-based genome engineering. Particularly, due to an important fact, the general risk profile of CRISPR experimentation in human beings remains unknown and it is the scientific duty to incur in these situations and to evaluate them objectively, eliminating dogmas, misperceptions, and personal prejudices, but always accompanied by institutional observation and with bioethical limits well established by specialized committees (Gil, 2019; Greely, 2019).

The great potential of genetic modification by means of CRISPR Cas, of cells or tissues, even of whole organisms, raises questions about its feasibility, since the modification does not remain only in the modified organism, but also in its offspring. The main argument of legalization can be divided into two main currents, the one that calls for laws that regulate the obtaining of patents to make these technologies accessible to patients who require it and those that regulate the use under controlled conditions, and the one where governments should be aware of who is developing studies about it. The latest due to the emergence in recent years of the so-called biohackers (Landrain, 2013; de Lecuona et al., 2017).

### Biohackers

Biohacker communities have proliferated in the world, without anyone being able to stop them, who are dedicated to research, development, and innovation of all kinds of scientific and technological activities. These communities are dedicated to exceeding the limits of biology, arguing that pharmaceutical companies are enriched by the development of techniques that could be performed at home without any problem, without clinical control, and without medical supervision, in addition to the affordable cost. Its main task is to generate treatments to increase life, cure diseases, increase available treatments, and increase the physical, biological, and physiological capacities of humanity (Kera, 2012; Meyer, 2012; Landrain, 2013; Gil, 2019).

These communities support the fundamental ethical arguments, adding them to philosophical theories that had not even been touched in recent years. At the time of the alchemists, it was only intended to stop the deterioration of age, but nowadays the cure of genetic and motor diseases is sought, with the argument of increasing human capacity within the framework of freedom, through DIYBio or Do It Yourself Biology. The topic of DIYBio became relevant since 2005, when it was mentioned at CodeCon, the possibility of purifying DNA at home with simple objects led to the promotion of free research in DNA biology and, together with various ethical controversies on the synthetic biology, the modification of eukaryotic organisms, with the problem of having information on biological techniques freely accessible, without legal regulation, control, and validation of a scientific organism (Meyer, 2012; Landrain, 2013; Gil, 2019).

In the United States Federal Drug Administration (FDA) has detected the presence of biohackers in its territory, they have experimented with various treatments from their own garage, highlighting the intradermal injection of DNA molecules modified by CRISPR Cas, that promise to cure a disease. The persecution of biohackers in the USA has been given for the practice of medicine without a license, however, these individuals could be accused of misuse of medical treatment, but to date in North American laws, the CRISPR Cas is not considered medical treatment, for which these acts could not be condemned, which results in the importance of countries, including the World Health Organization, of the guidelines for the international regulation of the use of CRISPR Cas as medical treatment and who, how, and where these biological technologies can be developed (Delfanti, 2011; Hirsch et al., 2019).

## CRISPR-Cas Modification, Legal Regulation

Since 1975, at the Asilomar conference, a growing concern was expressed about the use of recombinant DNA, despite the fact that the use of technology applied to DNA was allowed, the bioethical arguments regarding the application of genetic engineering to humanity continues to be the subject of deep debate (Evitt et al., 2015; Capella, 2016; Brokowski and Adli, 2019). In the 1960s, the theory of gene therapy in Stanfield’s experimental trial, referring to congenital metabolic diseases, was questioned regarding the ethical problems surrounding its execution. Until 1990, gene therapy was approved in humans, at least at the subclinical level, in such a way that it was confirmed in the Whiley Database on Gene Therapy Clinical Trials Worldwide, of the National Institute of Health (NIH) of the United States. However, until 2000 there were indications of the reliability of these therapies in humans, after arduous attempts to improve the technique for the treatment of severe combined adenosine deaminase immunodeficiency (ADA-SCID) (Espinosa and Hernández-Hernández, 2020).

Genetic modification is a well-known topic for bioethics, which is far from being forgotten and perhaps further from being resolved. The first issue in question is whether to allow the use of CRISPR Cas technology for gene modification, since it is doubtful that it can be put into practice in humans. Various statements around the world have expressed their concern about the regulation of gene editing. In the United Kingdom in 2015, the meeting of the Hinxton Group and the international meeting of the Academy of Sciences of the United States of North America took place, arguing that for no reason should clinical research applied to the clinic be limited, due to the concern of misuse. With the limitations imposed by bioethics in gene editing in embryos, the experiments were allowed by the Authority for Human Embryology and Fertilization of the United Kingdom, with the controversy crossed over the generation of humans modified by CRISPR Cas with resistance to HIV (Capella, 2016; Gamboa-Bernal, 2016; Hirsch et al., 2019).

Currently, Mexico seems to be a cradle for the development of stem cell treatments; nevertheless, the growth in the availability of these treatments makes Mexico a destination susceptible to bioethical conflicts due to the relatively easy obtaining of unproven applications, given the lack of scientific and legal regulation. Research with humans or tissues from humans in Mexico is governed by the General Health Law and the Regulation of the Law on Research Matters, it includes the Official Mexican Standard NOM-012-SSA3-2012, and the Declaration of Helsinki, which together provide the statutes by which the institutional Bioethics committees analyze the approval or prohibition of preclinical and clinical studies where humans are studied. The restrictions and limitations that researchers will have in this matter are listed in international treaties (López-Pacheco et al., 2016; Espinosa and Hernández-Hernández, 2020).

The genetic modification techniques based on CRISPR Cas are extremely novel, due to their qualities and relative ease of design, but not of execution, which allows the genetic modification of humans. The arguments that are presented against its use are valid, due to the fact of the possible alteration of the physiology of the organism, without ceasing to consider it beneficial if a health problem is corrected. Havoc or damage could translate into long-term damage that, translated into evolutionary events, would be a catastrophic event if not taken seriously. Alternatively, the concern of the scientific community focuses on the fact that there are no real limits for scientists, from a legal point of view, therefore, it is not clear how far the power of genetic modification can go and the weight that has on bioethical practices that seek to always go toward respecting life and the rights of living beings, in addition to the implementation of the four principles of bioethics. The scrutiny of current knowledge of these technologies, the way that these can either help or fail to achieve desired modifications, and the future promise and challenge of therapeutic genome editing, should be open for discussion, not only by scientists and physicians, in order to overcome the problem of Techno-Scientific Colonialist Paternalism (Arguedas-Ramírez, 2020).

We should consider the insight given by bioethicists in this subject, to mediate and be aware of the pros and cons of these new technologies, highlighting the importance of an open public discussion in which both parties are taken into consideration: the scientific and ethical facts to define the real issues in this picture. The role of the government in these regulations and instances to be addressed, should be to regulate and make sure to satisfy the needs to benefit all the society in need of these technologies, not only the privileged population. The latter, to ensure the ethical use of these systems to try to reduce gaps and social inequalities instead of opening new ones, while considering the current sociopolitical, economical, and historical issues, such as the anti-scientific movements and the politicization of science (Arguedas-Ramírez, 2020).

Therefore, the content of the current paper relies on opening a discussion in which the currently known real issues surrounding these technologies are described, in order to overcome the fear and doubts that can be generated in the global population.

### Future Perspectives of CRISPR Cas

The use of CRISPR Cas has generated an enormous progress in the development of biotechnology, it is the most outstanding discovery of the 21st century. The vision for the future that is expected from the CRISPR Cas system, from an anthropocentric position, is the cure of rare or catastrophic diseases such as cancer, diabetes, or congenital anemia, among others. In the health area, the application of this system would allow the fight against diseases such as HIV, malaria, dengue, Zika virus or the current SARS-CoV-2. In addition to the above, there is the possibility of interrupting the advance of bacterial resistance to antibiotics, in addition to decreasing the virulence of bacterial isolates that cause infections. The purpose is to always try to help human health, but care must be taken not to cause unwanted alterations in the patient (Escalona-Noguero et al., 2021; Tsou et al., 2021; Yadav et al., 2021).

However, the area of application of this system is enormous, agriculture should benefit from the CRISPR Cas helping to improve food and increasing availability to combat famine and food shortages. In the biotechnology industry, the CRISPR Cas suggests being valuable for the implementation or modification of metabolic routes, this would increase the yields in obtaining the product, optimizing the processes for obtaining products of biological origin (Nidhi et al., 2021).

The use of CRISPR Cas implies legal and bioethical principles, initially these principles should protect human dignity, safeguard the integrity of the patient, and safeguard the content of their genetic information to avoid inappropriate uses. Another aspect that should be considered is solving the inherent risk of undergoing these treatments that will not show adverse effects, if any, in the short-term. Together, bioethics and legal law will have to work together to regulate the use of patient information, protecting fundamental rights, such as health. Considering that these techniques must be carefully evaluated and observed, not to prohibit them, but to handle them with care, because it is known that with this technique some diseases could be eradicated (Nidhi et al., 2021).

## Author Contributions

LG-A, CH-C, and GC-E developed the structural design of the review and drafted the manuscript. LG-A, JV-L, LP-R, OC-M, CH-C, and GC-E reviewed the manuscript critically for important intellectual content and appropriate academic content. All authors read and approved the final manuscript.

## Conflict of Interest

The authors declare that the research was conducted in the absence of any commercial or financial relationships that could be construed as a potential conflict of interest.
